# Definition of Critical Periods for Hedgehog Pathway Antagonist-Induced Holoprosencephaly, Cleft Lip, and Cleft Palate

**DOI:** 10.1371/journal.pone.0120517

**Published:** 2015-03-20

**Authors:** Galen W. Heyne, Cal G. Melberg, Padydeh Doroodchi, Kia F. Parins, Henry W. Kietzman, Joshua L. Everson, Lydia J. Ansen-Wilson, Robert J. Lipinski

**Affiliations:** 1 Department of Comparative Biosciences, School of Veterinary Medicine, University of Wisconsin-Madison, Madison, WI, United States of America; 2 Molecular and Environmental Toxicology Center, School of Medicine and Public Health, University of Wisconsin-Madison, Madison, WI, United States of America; Pennington Biomedical Research Center/LSU, UNITED STATES

## Abstract

The Hedgehog (Hh) signaling pathway mediates multiple spatiotemporally-specific aspects of brain and face development. Genetic and chemical disruptions of the pathway are known to result in an array of structural malformations, including holoprosencephaly (HPE), clefts of the lip with or without cleft palate (CL/P), and clefts of the secondary palate only (CPO). Here, we examined patterns of dysmorphology caused by acute, stage-specific Hh signaling inhibition. Timed-pregnant wildtype C57BL/6J mice were administered a single dose of the potent pathway antagonist vismodegib at discrete time points between gestational day (GD) 7.0 and 10.0, an interval approximately corresponding to the 15^th^ to 24^th^ days of human gestation. The resultant pattern of facial and brain dysmorphology was dependent upon stage of exposure. Insult between GD7.0 and GD8.25 resulted in HPE, with peak incidence following exposure at GD7.5. Unilateral clefts of the lip extending into the primary palate were also observed, with peak incidence following exposure at GD8.875. Insult between GD9.0 and GD10.0 resulted in CPO and forelimb abnormalities. We have previously demonstrated that Hh antagonist-induced cleft lip results from deficiency of the medial nasal process and show here that CPO is associated with reduced growth of the maxillary-derived palatal shelves. By defining the critical periods for the induction of HPE, CL/P, and CPO with fine temporal resolution, these results provide a mechanism by which Hh pathway disruption can result in “non-syndromic” orofacial clefting, or HPE with or without co-occurring clefts. This study also establishes a novel and tractable mouse model of human craniofacial malformations using a single dose of a commercially available and pathway-specific drug.

## Introduction

Orofacial clefts (OFCs), including cleft lip with or without cleft palate (CL/P) and cleft palate only (CPO), are commonly occurring human birth defects that cause significant morbidity and require extensive medical intervention. Non-syndromic CL/P and CPO are genetically complex traits. Inheritance patterns are non-Mendelian and in most cases, affected patients have no positive family history [1–3]. This suggests that OFCs result from interacting genetic and environmental factors. However, basic research efforts to elucidate the complex etiology and pathogenesis of OFCs have been hampered by a paucity of faithful and tractable animal models [4].

The Hedgehog (Hh) signaling pathway is required for development of the brain and face. In the developing brain, *Sonic Hedgehog* (*Shh*) is detected in the neuroectoderm of the ventral mesencephalon, with expression then expanding rostrally into the diencephalon and telencephalon [5, 6]. Along with its well-described role in neurospecification, expression of *Shh* in the forebrain neuroectoderm is required for patterning and expansion of the adjacently developing midface [7, 8]. Pathway activity is detected in the medial nasal processes that contribute to the median aspect of the upper lip and primary palate, as well as the maxillary processes that give rise to the lateral aspects of the upper lip and the secondary palate [9, 10].

Chemical and genetic perturbation of the Hh pathway is classically associated with holoprosencephaly (HPE), a condition defined by incomplete division of the medial forebrain, which commonly co-occurs with OFCs [11]. Human genetic analyses, as well as transgenic and teratogenic animal models, have also implicated Hh pathway disruption in the pathogenesis of OFCs [12–14]. For example, we previously demonstrated that *in utero* exposure to the natural Hh pathway antagonist cyclopamine in the mouse causes CL/P that co-occurs with subtle forebrain abnormalities, but not the defining characteristics of HPE [15–17]. Disruption of the Hh pathway has also been shown to cause CPO in transgenic mouse models [12, 13]. A cohesive mechanism for the causation of these related but distinct birth defects by disruption of a single signaling pathway has yet to be established.

Recognizing the dynamic role of Hh signaling in multiple spatiotemporally-dependent aspects of brain and face development, the studies described here were directed at defining the stage-specific outcomes of Hh pathway inhibition. Precise stages of embryonic development were targeted by acute exposure to the synthetic Hh pathway antagonist vismodegib, which acts through the same mechanism as cyclopamine but is significantly more potent [18]. For comparison to our previously established model of CL/P, an additional cohort of animals was exposed to cyclopamine over a less acute period of embryogenesis. Resultant phenotypes were methodically examined with a focus on clinically-relevant dysmorphology of the brain and face.

## Materials and Methods

### Timed mouse mating

This study was carried out in strict accordance with the recommendations in the *Guide for the Care and Use of Laboratory Animals* of the National Institutes of Health. The protocol was approved by the University of Wisconsin School of Veterinary Medicine Institutional Animal Care and Use Committee (protocol number 13–081.0). C57BL/6J mice were purchased from The Jackson Laboratory and housed under specific pathogen-free conditions in disposable, ventilated cages (Innovive, San Diego, CA). Rooms were maintained at 22 ±2 degrees Celsius and 30–70% humidity on a 12 hour light, 12 hour dark cycle. Mice were fed 2920x Irradiated Harlan Teklad Global Soy Protein-Free Extruded Rodent Diet. For timed matings, 1–3 not previously pregnant female mice were placed with a single male for 1–2 hrs and subsequently examined for the presence of copulation plugs. The beginning of the mating period was designated as gestational day (GD)0.

### Hh antagonist exposure

Vismodegib (Toronto Research Chemicals) was suspended as 3mg/ml in 0.5% methyl cellulose (Sigma) with 0.2% Tween (Sigma). Individual suspensions were prepared within 30 minutes of administration. Pregnant mice were administered 40mg/kg vismodegib (aka GDC-0449) by oral gavage at indicated time points (± 20 minutes), including: GD7.0, 7.25, 7.5, 7.75, 8.0, 8.25, 8.5, 8.625, 8.75, 8.875, 9.0, 9.25, 9.5, 9.75, and 10.0. This dosing regimen has previously been shown to effectively inhibit Hh signaling activity in a mouse xenograft tumor model [19]. That same study demonstrated that the maximum serum concentration of vismodegib was reached one hour after administration of a 50mg/kg dose, which yielded a serum half-life of 3.9 hours. For examination of palatal shelf morphogenesis, timed-pregnant mice were administered 80mg/kg vismodegib by oral gavage at GD9.75 to increase CPO penetrance. All vismodegib doses were administered during the light cycle between 8am and 6pm. Cyclopamine was administered at 120mg/kg/d via subcutaneous infusion from GD8.25 to ~9.375 using microosmotic pumps (Alzet model 2001D) as described previously [15, 17]. For each vismodegib and cyclopamine exposure paradigm, 5–7 litters were examined. The control group consisted of fetuses exposed to 0.5% methyl cellulose with 0.2% Tween by oral gavage at GD7.75, 8.875, or 9.5. 5 litters were collected for each vehicle exposure paradigm.

### Dissection and imaging

Pregnant dams were euthanized at GD17 ± 2 hours by CO_2_ asphyxiation and subsequent cervical dislocation. Crown-rump length was measured at time of dissection. Fetal specimens were fixed in 10% formalin for at least 1 week prior to imaging. Images of the whole body, head, and palate were captured using a MicroPublisher 5.0 camera connected to a Nikon SZX-10 stereomicroscope.

### Phenotypic assessment

Randomly labeled images were assessed by a single rater blinded to treatment. The rater scored each fetus for the presence of HPE, OFCs (noting the extent of the cleft), kinked tail, edema, and overt mandibular hypoplasia. HPE was classified by the presence of specific facial features previously described to be predictive of defining brain abnormalities in GD17 mouse fetuses [20]. Specifically, animals exhibiting loss of the medial lip notch with diminished or absent pigment at the tip of the nose, or having a single nostril (cebocephaly) were classified as HPE. Animals exhibiting a diminished area of pigmentation below the nose and/or deficient but present medial lip notch were classified as having midfacial hypoplasia without HPE. The predictive accuracy of this approach was confirmed by histological analysis of animals classified with midfacial hypoplasia versus HPE (S1 Fig.). Fetuses with HPE co-occurring with median cleft lip and/or cleft of the secondary palate were classified as HPE and not included in CL/P or CPO categories. Additionally, a small number of animals exposed between GD8.0 and 9.0 exhibited secondary palate clefts co-occurring with overt mandibular hypoplasia. Appearing to represent two infrequent and distinct mechanisms, these animals were not included in the CPO classification but are enumerated in parentheses in **S1 Table**.

### Bone & cartilage staining

Fetal specimens were skinned, eviscerated and fixed in 95% or 100% ethanol for at least 1 week, then placed overnight in a staining solution containing 8ml of 100% ethanol, 10ml of acetic acid, and 2ml of 1% alcian blue/3% acetic acid (Poly Scientific). They were then rinsed twice for one hour and subsequently left overnight in 100% ethanol. Following clearing for two hours in 1% potassium hydroxide, staining in 0.005% alizarin red (Bio World) in 2% potassium hydroxide for four hours was performed. Fetuses were then rinsed in 2% potassium hydroxide once quickly, once for one hour, and then left overnight in 1% potassium hydroxide. Finally, they were transferred to 1:3 glycerol in 2% potassium hydroxide for eight hours and stored and imaged in a 1:1 solution.

### Histology

GD17 fetuses were fixed in Bouin’s solution (Sigma-Aldrich) for at least one week, and then transferred to 70% ethanol. Following paraffin embedding, 10μm sections were produced and stained with Hematoxylin and Eosin by standard protocols.

### Palatal shelf assessment

Embryos exposed to cyclopamine as described above, 80mg/kg vismodegib at GD9.75, or vehicle alone at GD9.75 were harvested at GD14.5. Cuts between the upper and lower jaw were made with a scalpel to expose the roof of the oral cavity, which was first imaged by light microscopy as described above. From these images, linear measurements of palatal shelf length and width were performed using Adobe Photoshop v14.1.2. Unilateral measurements of palatal shelf width were made at 1/3 of the shelf length from the most rostral aspect. Fetuses were excluded from this analysis if connective tissue obstructed clear ascertainment of palatal borders, or if the initial cut removed palatal tissue from both shelves. For each treatment group, average length and width were compared via a one-way ANOVA followed by Holm-Sidak's multiple comparison test using Graphpad Prism software (v6.04). An alpha value of 0.05 was maintained for all analyses. Tissue was then processed and imaged by scanning electron microscopy as previously described [21].

### Supporting Information

#### Measurement of midfacial width

From the facial images of GD17 fetuses, linear measurements of snout width between the most lateral, 3^rd^ row of vibrissae, as previously described [22], were produced in Adobe Photoshop v14.1.2. Measurements were performed on all fetuses with HPE resulting from vismodegib exposure at GD7.75, those with CPO resulting from vismodegib exposure at GD9.5, and those with CL/P resulting from cyclopamine exposure. These values were compared to those of a control group, comprised of fetuses exposed to vehicle at GD7.75. For each treatment group, measurements were compared via a one-way ANOVA followed by Holm-Sidak's multiple comparison test using Graphpad Prism software (v6.04). An alpha value of 0.05 was maintained for all analyses.

#### Fetal sex determination

Fetal sex was determined by dissection and visual identification of ovaries or testes. Statistical analysis of potential differences in incidence of HPE, CL/P and CPO between sexes was performed. Included in the analysis were animals with vismodegib-induced HPE and CPO, as well as cyclopamine-induced CL/P. Male-female sex ratios of affected animals were determined for each litter. Potential differences between sexes were examined by chi-squared goodness of fit test. Data entry and subsequent calculations were performed in Microsoft Excel 2013.

## Results

### Stage of exposure-dependent facial dysmorphology

**Fig 1:**
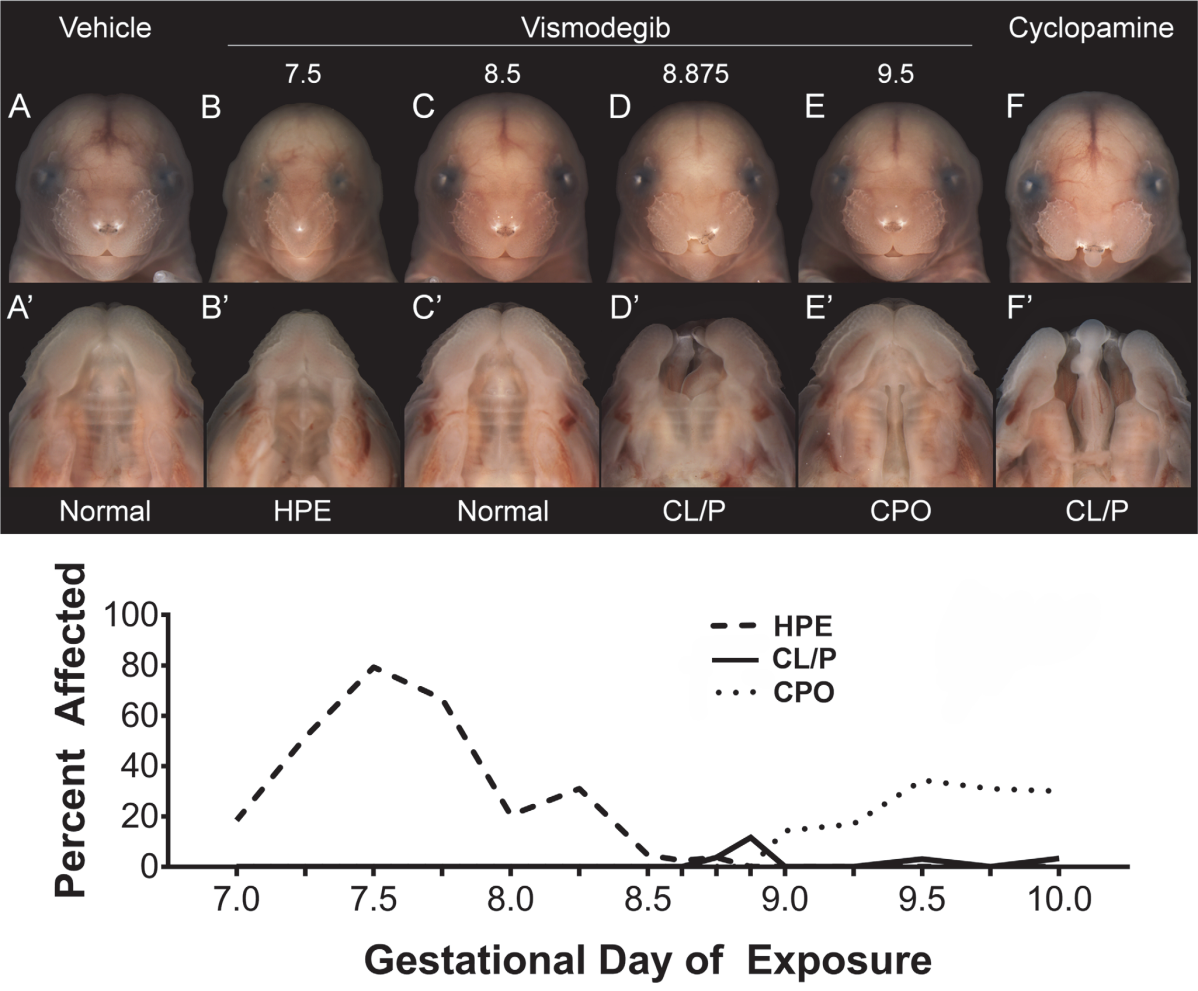
Stage of exposure-dependent facial dysmorphology. Single doses of vismodegib were administered at discrete time points indicated by tick marks on the x-axis, including: GD7.0, 7.25, 7.5, 7.75, 8.0, 8.25, 8.5, 8.625, 8.75, 8.875, 9.0, 9.25, 9.5, 9.75, and 10.0. Cyclopamine was administered by subcutaneous infusion from GD8.25 to ~9.375. Representative examples of distinct face and palate phenotypes are shown, including apparently normal (Normal), HPE, CL/P, and CPO. Note that lateral lip clefts resulting from acute vismodegib exposure typically extended into the primary palate (D’), while those resulting from cyclopamine exposure extended into both the primary and secondary palate (F’). The penetrance of HPE, CL/P, and CPO phenotypes resulting from stage-specific vismodegib exposure is shown in the graph. 5–7 litters were examined for each exposure permutation.

Acute administration of 40mg/kg vismodegib between GD7.0 and 8.25 caused craniofacial dysmorphology indicative of HPE, including microcephaly, marked mid-facial hypoplasia, hypotelorism, a highly arched palate, and in severe cases cebocephaly (Fig. 1B,B’). Peak incidence of HPE-associated craniofacial dysmorphology was caused by exposure at GD7.5. Lateral cleft lip, in most cases extending into the primary but not secondary palate, resulted from later exposure with peak incidence following administration at GD8.875 (Fig. 1D,D’). Vismodegib exposure between GD9.0 and 10.0 caused clefts of the secondary palate only (Fig. 1E,E’), with peak incidence resulting from administration at GD9.5. In these animals, CPO was observed in the absence of other gross abnormalities of the face including the upper lip, primary palate, and mandible. As described previously, cyclopamine exposure from GD8.25 to ~9.375 by subcutaneous infusion caused lateral clefts of the lip extending into the primary and secondary palate (Fig. 1F,F’). The maximum penetrance of vismodegib-induced HPE (77%) was considerably higher than that for CL/P (13%) and CPO (34%). However, a high penetrance of CL/P (81%) was observed following less-acute cyclopamine exposure. Inter- and intra-litter penetrance is shown in S2 Fig. Less frequently observed phenotypic variants of HPE, CL/P, and CPO resulting from each exposure paradigm are shown in S3 Fig. The majority of fetuses exposed to vismodegib between the critical periods for HPE and OFCs exhibited grossly normal craniofacial morphology (Fig. 1C,C’). For each treatment group the number of litters and fetuses examined, average litter size, average crown-rump length, and incidence of each of the phenotypes listed above are presented in S1 Table

### Phenotypic characterization of HPE, CL/P, and CPO

**Fig 2:**
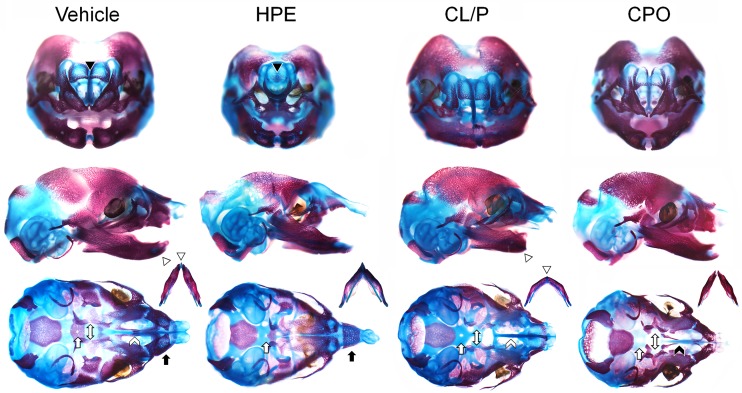
HPE, CL/P, and CPO associated craniofacial abnormalities. Bone and cartilage are stained red and blue, respectively. Top row shows coronal view, middle row shows lateral view, and bottom row shows inferior view with mandibles removed and shown to the right. Animals with HPE exhibit a single, small nasal bone (black arrowhead) and a fused premaxilla (black arrow). Animals with CL/P and CPO have increased width between pterygoid and palatine bones compared to vehicle control (white double arrow), and an absent basisphenoid bone, while in those with HPE only the anterior half is ossified (white arrow). Compared to vehicle control, animals with CL/P have shorter mandibles (white arrowhead) and absent vomer and palatal premaxilla processes (white caret). In animals with CPO the vomer is displaced posteriorly (black caret).

Bone and cartilage staining was performed to further characterize Hh antagonist-induced HPE, CL/P, and CPO phenotypes (Fig. 2). Animals with HPE displayed morphology consistent with a loss of midline, including a single fused premaxilla, single small nasal bone, single nasal capsule, and apparent absence of vomer bone, accompanied by maxilla and palatine bones that were too closely approximated. Additionally, the anterior portion of the basisphenoid appeared normal, yet the posterior portion was not ossified. Hard tissue malformations in animals with cyclopamine-induced CL/P included absent basisphenoid, presphenoid and vomer bones, and the palatal processes of the premaxilla. The premaxilla appeared deficient, and the maxilla, pterygoid, and palatine bones were too widely spaced. Consistent with described clinical phenotypes, animals with CL/P also exhibited mandibular dysmorphology [23]. As with CL/P, animals with CPO had absent basisphenoid bones. The inferior aspect of the premaxilla was deficient, while the lateral aspect appeared normal. The presphenoid appeared divided, and the palatine, maxilla, pterygoid, and vomer bones were too widely spaced. The posterior vomer did not contact the maxilla, but instead was oriented posteriorly toward the base of the skull.

**Fig 3:**
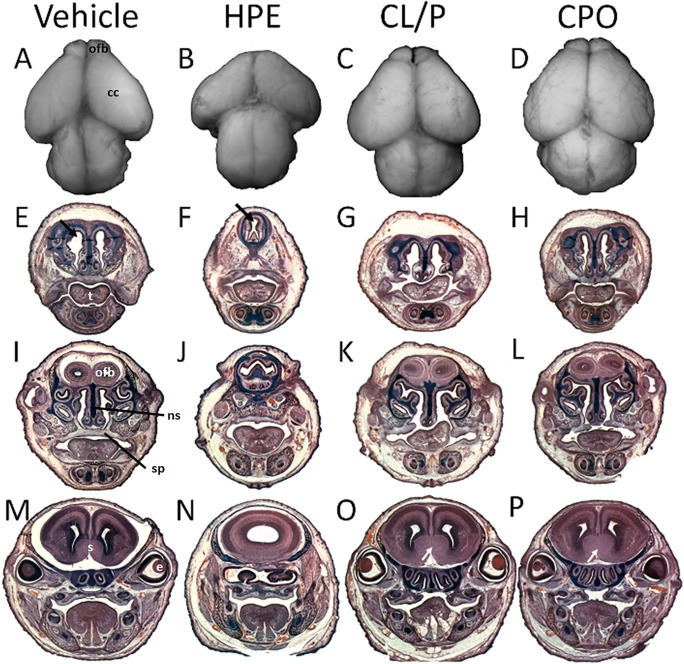
HPE, CL/P, and CPO associated brain morphology. Superior views of dissected brains are shown for a vehicle-exposed normal animal (A) and for representative examples of animals with vismodegib-induced HPE (B), cyclopamine-induced CL/P (C), and vismodegib-induced CPO (D). Severe hypoplasia of the cerebral cortices (cc) and olfactory bulb (ofb) absence is apparent in the animal with HPE. In animals with CL/P and CPO, the cerebral cortices appear to be of approximately normal size but the olfactory bulbs are hypoplastic. Serial coronal sections of comparably classified animals are shown in E-P. Notable HPE-associated features include a single central nasal passage with nasal septum (ns) cartilage absence (black arrow), olfactory bulb agenesis (J), and a single telencephalic vesicle (N). Grossly normal division of the olfactory bulbs (K, L) and cerebral cortices (O, P), and apparent forebrain septal (s) region hyperplasia (white arrows) is observed in animals with CL/P and CPO. (t) Tongue, (e) eye, (sp) secondary palate.

Next, brain morphology associated with HPE, CL/P, and CPO was examined by gross dissection and histological analysis (Fig. 3). As expected, marked medial forebrain deficiency was observed in animals with facial features typical of HPE. Those with the most severe midfacial deficiencies exhibited a single telencephalic vesicle with olfactory bulb aplasia. Animals with cyclopamine-induced CL/P and vismodegib-induced CPO exhibited olfactory bulb hypoplasia and septal region hyperplasia but otherwise grossly normal forebrain morphology, including complete division of the cerebral cortices.

### Morphogenesis of secondary palate clefts

**Fig 4:**
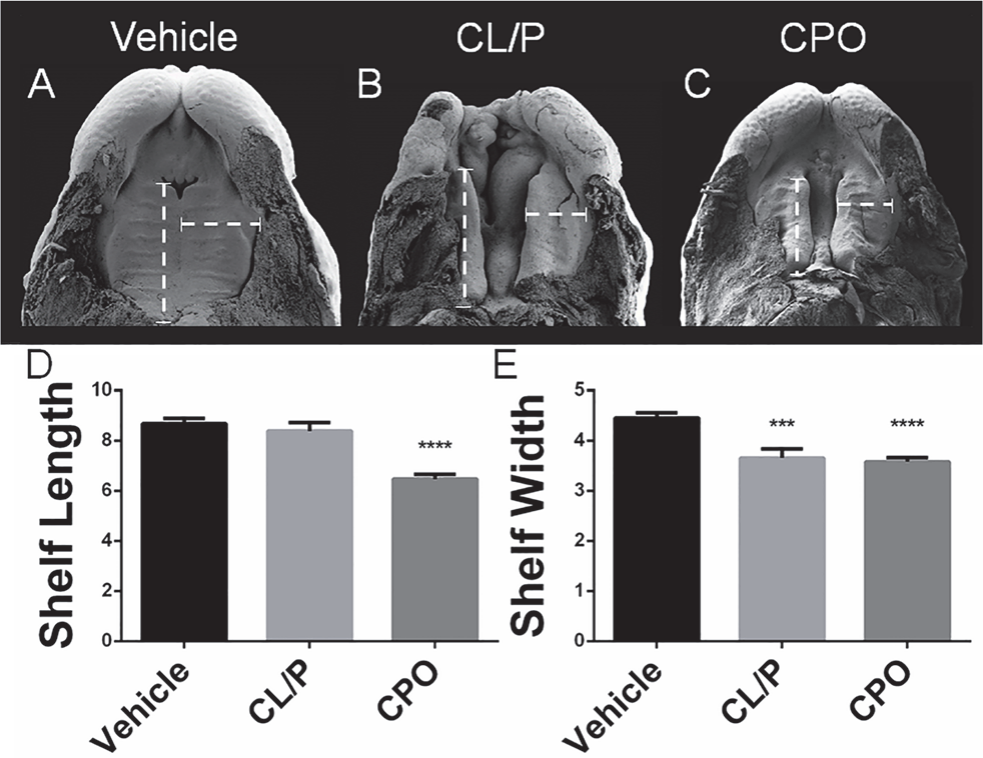
CL/P and CPO associated secondary palate morphology. In vehicle-exposed embryos at GD14.5 (A) the secondary palatal shelves have approximated and made contact at the midline. In affected cyclopamine-exposed embryos with cleft lip (B), palatal shelves are widely spaced and deficient in width. In vismodegib-exposed embryos (C), secondary palatal shelves have also elevated but are deficient in both length and width. Length (D) and width (E) measurements (arbitrary units), as depicted by the dashed calipers, were made on light microscopy images (S4 Fig.). Shelf width was determined at 1/3 shelf length from the most rostral aspect. *** p<0.001, **** p<0.0001

We next examined the morphogenesis of secondary palate clefts associated with CL/P versus CPO. In addition to morphologic assessment, length and width measurements (S4 Fig.) were made of secondary palatal shelves at GD14.5. In the majority of vehicle-exposed embryos (82%), the median aspect of the opposing shelves had begun to make contact at the midline (Fig. 4A). The palatal shelves of cyclopamine-exposed embryos with cleft lip did not make contact at the midline and were deficient in width (Fig. 4B,E). Vismodegib-exposed embryos exhibited a significant deficiency of the secondary palatal shelves in both length and width dimensions (Fig. 4C,D,E). As a possible alternative mechanism for secondary palate clefts, midfacial width was measured in affected GD17 fetuses. While increased midfacial width was observed in animals with CL/P [16], those with CPO exhibited a slight but significant decrease compared to vehicle (S5 Fig.).

### Additional stage of exposure-dependent phenotypes

**Fig 5:**
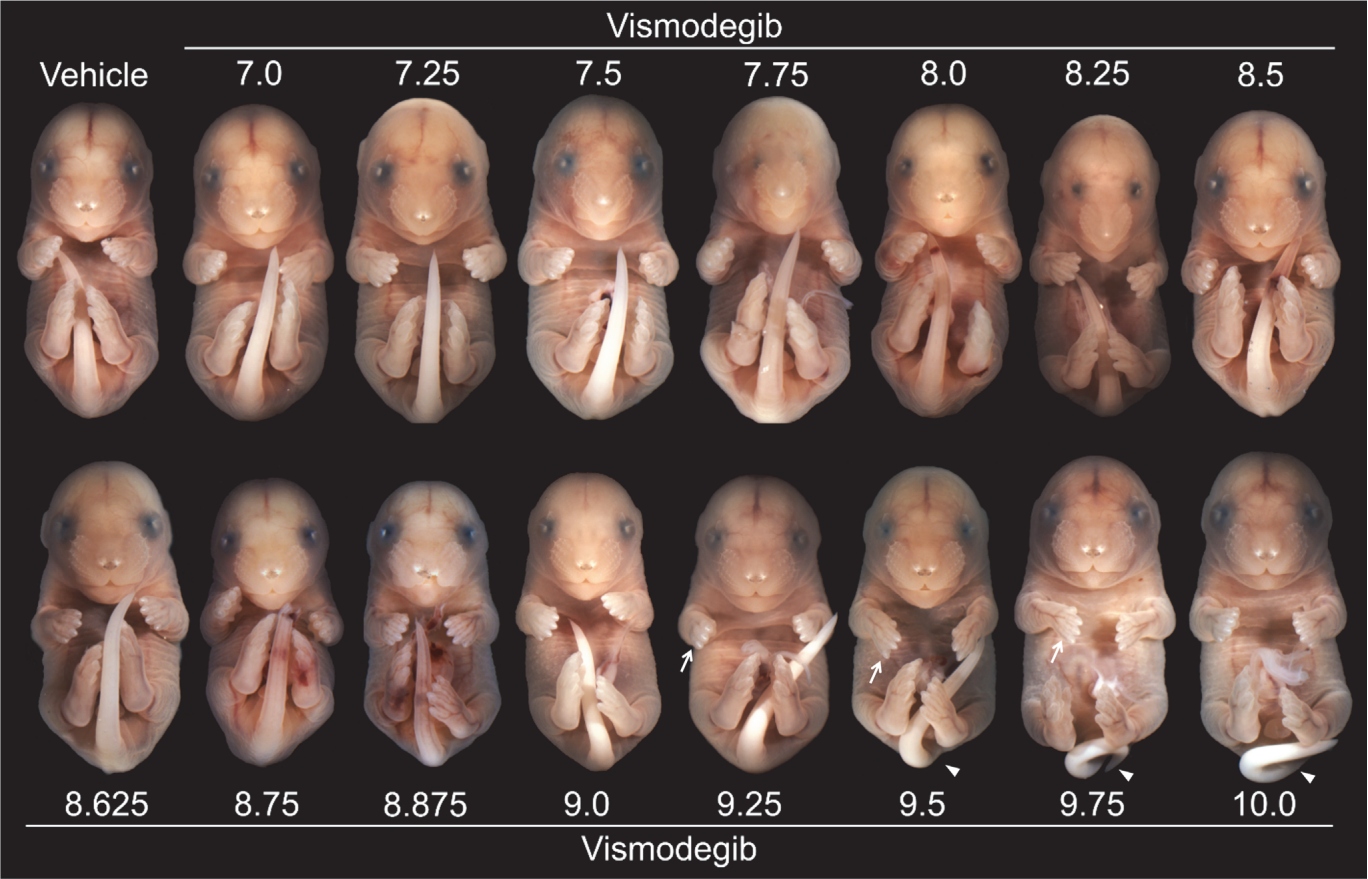
Additional stage of exposure-dependent phenotypes. Along with a vehicle-exposed control (A), representative examples of phenotypic outcomes are shown with numbers indicating the gestational stage of acute vismodegib administration. Later exposure was associated with forelimb ectrodactyly, as exhibited bilaterally in fetuses exposed from 9.25 to 9.75 (arrows point to absent fifth digits on the right limb). Kinked tail phenotypes were caused by exposure between GD9.5 and 10.0 (arrowheads). Edema is also apparent in fetuses exposed at GD9.75 and 10.0. For each treatment group the number of litters and fetuses examined, mean litter size and crown-rump length, and the incidence of edema, forelimb ectrodactyly, and kinked tail defects are presented in S1 Table.

In addition to facial and brain dysmorphology as described above, phenotypic assessment conducted on GD17 fetuses revealed malformations involving the limbs and vertebrae (Fig. 5). Vismodegib exposure between GD9.0 and GD10.0 was associated with forelimb ectrodactyly, including a bilateral loss of one or two posterior digits in affected animals (Fig. 5, S6 Fig.). Kinked tail phenotypes were caused by exposure between GD9.5 and 10.0 (Fig. 5), suggesting a disruption of vertebral development that may be secondary to abnormal neurulation [24]. Bone and cartilage staining revealed focal vertebral anomalies, localized from cervical to lumbar regions with increasing stage of exposure (S6 Fig.). For example, HPE-associated exposure at GD7.75 caused fusion of cranial cervical vertebrae. Later CPO-associated exposure at GD9.5 was associated with abnormal lumbar cartilage development and deficient vertebral body ossification.

## Discussion

Perturbation of the Hh signaling pathway has been associated with HPE, CL/P and CPO in both animal models and in clinical populations. In humans, *SHH* mutations are associated with HPE with or without co-occurring OFCs, or facial dysmorphology in the absence of detectable neuroanatomical anomalies [25]. Transgenic mouse models and clinical case reports have also linked genetic disruption of the Hh pathway to both CL/P and CPO in the absence of HPE [12, 14, 17]. Here we demonstrate that each of these birth defects can result from acute Hh pathway inhibition, with the specific outcome precisely dependent upon the timing of insult. Consistent with the finding that temporally-dependent cyclopamine administration elicits the spectrum of HPE in the chick [26], the findings described here in the mouse have more direct implications for human health, particularly in regard to advancing our understanding of the complex etiopathogenesis of CL/P and CPO. Importantly, we show that the critical period for each of these outcomes is between GD7.0 and 10.0 in the mouse. This interval approximately corresponds to the 15^th^ to 24^th^ days of human gestation, when many pregnancies are not yet recognized.

HPE occurs in approximately 1 in 10,000 live births, yet an observed prevalence of 1 in 250 conceptuses suggests that it is one of the most common human developmental abnormalities [27, 28]. OFCs represent the second most prevalent human birth defect, affecting 1–2/1,000 newborns [1]. While CL/P and CPO have traditionally been considered causally and pathologically distinct, evidence from both human pedigrees and animal models illustrate several exceptions to this rule [29, 30]. Approximately 50% of CPO and 70% of CL/P cases are considered “non-syndromic.” Among these, clefts of the lip extending into the primary palate and secondary palate are twice as common as those affecting only the lip and primary palate [31]. The data presented here argue that the critical period for CL/P in the mouse is much narrower than that for HPE and CPO (Fig. 1). Consistent with that premise, subcutaneous infusion of cyclopamine yielding a serum steady state concentration from approximately GD8.5 to 9.375 [15] resulted in cleft lip with near complete penetrance. In humans, the prevalence of OFCs differs by race and sex, with CL/P more common in males and CPO more common in females [1]. Sex differences were not observed in animals with vismodegib-induced HPE or CPO, or those with cyclopamine-induced CL/P (S7 Fig.).

We have previously demonstrated that cyclopamine-induced cleft lip results from a tissue deficiency of the lower aspect of the medial nasal process [16]. Normally, this tissue unites with the rostral aspect of the maxillary process (maxillary prime) to close the upper lip. The secondary palate develops from maxillary-derived outgrowths that elevate above the tongue, approximate, and fuse at the midline. Anteriorly, the secondary palatal shelves fuse with the medial nasal process-derived primary palate. Multiple mechanisms have been implicated in the morphogenesis of secondary palate clefts. We previously proposed that this defect results from increased midfacial width associated with cyclopamine-induced cleft lip. The data described here suggests that cyclopamine exposure spanning the critical periods for both cleft lip and CPO induces secondary palate clefts through a combination of increased midfacial width and subtle deficiency of the palatal shelves. The operation of dual mechanisms is supported by the finding that vismodegib-induced cleft lip typically extends into the primary but not the secondary palate.

We show here that Hh signaling antagonist-induced CPO is associated with deficiency of maxillary-derived secondary palatal shelves. While we did not specifically assess growth of the palatal shelves, we did find that CPO occurred without increased midfacial width, suggesting that the effect of vismodegib exposure in this window targets maxillary process morphogenesis. This observation is supported by the detection of pathway activity in the medial mesenchyme of the maxillary processes as early as GD9.5 [9]. A role for Hh pathway activity in later palatal shelf development has already been described. For example, conditional deletion of Smoothened after GD11.5 has been shown to result in decreased cell proliferation and decreased palatal outgrowth at GD13.5 [13]. The demonstration here that the critical period for Hh antagonist-induced CPO begins as early as GD9.0 suggests pathway sensitivity in the maxillary processes much earlier than previously recognized.

The Hh signaling pathway plays important roles in the patterning and growth of multiple tissues and organs, including the limbs and vertebrae [32, 33]. The finding described here that limb and vertebral anomalies co-occur with OFCs resulting from global Hh pathway antagonism is not surprising and likely has clinical relevance. While the mouse appears to be significantly more sensitive to teratogenic disruption of limb patterning (e.g. those resulting from prenatal ethanol exposure [34], both HPE and OFCs have been associated with forelimb malformations including ectrodactyly in clinical populations [35, 36]. Vertebral abnormalities have also been shown to preferentially co-occur with HPE and OFCs [37–41]. The variable co-occurrence of these abnormalities likely depends upon the spatiotemporal extent of the particular causative genetic or chemical insult.

Animal models have proven invaluable for elucidation of the etiopathogenesis of myriad diseases and developmental abnormalities. While murine and human craniofacial development is remarkably conserved [42], a paucity of faithful mouse models has long frustrated efforts to better understand the causes of OFCs [4]. The study described here addresses this need by demonstrating that HPE, CL/P and CPO in the mouse result from temporally-specific administration of vismodegib, a commercially available drug that potently inhibits Hh signaling without known off-target effects. Sensitive to both chemical and genetic disruption, future investigation of the Hh signaling pathway in the genesis of OFCs is a promising avenue to identify candidate human disease genes and culpable environmental agents.

## Supporting Information

S1 Fig Facial features accurately predict HPE. Relative to the vehicle-exposed control (A), a vismodegib-exposed fetus classified as having midfacial hypoplasia exhibits diminished area of pigmentation below the nose (arrowhead) and diminished but intact medial lip notch (B; arrow). Representative of those classified as HPE, the vismodegib-exposed fetuses shown in C and D exhibit loss of the medial lip notch with diminished or absent pigment at the tip of the nose, with the latter having a single nostril (cebocephaly). Histological sections from comparable animals show normal division of the cerebral cortices (cc) in animals classified as having midfacial hypoplasia, while those included as HPE demonstrate incomplete division of the cerebral cortices with communicating lateral ventricles. Note that the animal with HPE shown in (G) also has a secondary palate (sp) cleft. I-L show bone and cartilage staining in similarly classified animals, demonstrating a subtle midline deficiency in animals with midfacial hypoplasia, and a more severe phenotype in animals with HPE. White arrows show the premaxilla, which are fused in J, K and L. Arrowheads indicate the nasal bones, which are fused in K and L with an underlying single nasal capsule (L). (t) Tongue (e) eye.
(TIF)

S2 Fig Inter- and intra-litter penetrance. Vismodegib was administered at discrete time points indicated by tick marks on the x-axis including GD7.0, 7.25, 7.5, 7.75, 8.0, 8.25, 8.5, 8.625, 8.75, 8.875, 9.0, 9.25, 9.5, 9.75, and 10.0. Interlitter penetrance was determined by calculating the percentage of litters in which at least one affected fetus was observed. Intralitter penetrance was determined by calculating the percentage of affected fetuses within litters with at least one affected individual.
(TIF)

S3 Fig Phenotypic variants of HPE, CL/P and CPO. Face and palate dysmorphology infrequently co-occurring with HPE included exencephaly (A), secondary palate cleft (B, B’), median cleft lip (C, C’), or median cleft lip extending into the secondary palate (D, D’). Also infrequently, vismodegib exposure caused clefts of the secondary palate associated with severe mandibular hypoplasia (E, E’; arrow¬¬¬), cleft lip partially extending into the secondary palate, (F, F’), or incomplete cleft lip with secondary palate cleft (G, G’). Exposure during the critical period for CPO was also associated with incomplete fusion between the primary and secondary palate (H’ inset). Cyclopamine exposure infrequently caused unilateral clefts of the lip extending into the primary and secondary palate (I, I’), or midfacial hypoplasia with CPO¬ (J’).
(TIF)

S4 Fig Secondary palate morphology in CL/P versus CPO. Representative GD14.5 embryos exposed to vehicle at GD9.75 (A), cyclopamine (B), or vismodegib at GD9.75 (C). Vertical and horizontal dashed lines illustrate representative linear measurements of palate shelf length and width, respectively.
(TIF)

S5 Fig Midfacial width associated with CL/P versus CPO. Relative to vehicle control animals, snout width is increased in animals with cyclopamine-induced CL/P, but decreased in those with vismodegib-induced HPE and CPO. Values represent the mean + S.E.M. * p<0.05, **** p<0.001, compared to vehicle-exposed control group. Units are arbitrary.
(TIF)

S6 Fig Vismodegib-induced limb and vertebral abnormalities. Right forelimbs of animals with vismodegib-induced forelimb abnormalities (B,C) are shown with a vehicle-exposed control (A). Varying severity of ectrodactyly, including complete absence of the fifth digit (B) and the complete absence of the fifth and partial absence of the fourth digit (C). Relatively early treatment with vismodegib resulted in HPE with accompanying cervical spine malformations including fusion of the first and second vertebrae (D; black arrow). Later treatment with vismodegib caused CPO with abnormalities in the lumbar vertebrae and cartilage (E; white arrowhead). Additionally, absent ossification in the vertebral bodies (asterisk) is apparent. Bone and cartilage are stained red and blue, respectively.
(TIF)

S7 Fig Sex dependent-penetrance of HPE, CL/P, and CPO. To avoid litter bias, male-female sex ratios of affected animals were determined for each litter. Values represent the mean sex distribution for HPE, CL/P or CPO + SEM. Differences were not significant by chi-square goodness of fit test (p>0.05).
(TIF)

S1 Table Phenotypic assessment. 1 HPE co-occurring with median cleft lip (GD7.0, n = 1; GD7.25, n = 1; GD7.5, n = 2). 2 HPE co-occurring with exencephaly (GD7.0, n = 2; GD7.5, n = 1). 3 HPE co-occurring with secondary palate clefts. 4 Overt mandibular hypoplasia co-occurring with secondary palate clefts. * 0.005 ≤p<0.05 relative to vehicle-exposed controls as determined by a one-way ANOVA followed by Holm-Sidak's multiple comparison test using Graphpad Prism software (v6.04). ** p<0.005 relative to vehicle-exposed controls as determined by a one-way ANOVA followed by Holm-Sidak's multiple comparison test using Graphpad Prism software (v6.04). ^ 5 litters exposed to vehicle at GD7.75, 8.875, and 9.5 were collected, comprising the control group.
(XLSX)
