# Supplementary figures and images for: Definition of Critical Periods for Hedgehog Pathway Antagonist-Induced Holoprosencephaly, Cleft Lip, and Cleft Palate
Source: PLoS One. 2015 Mar 20;10(3):e0120517. doi: 10.1371/journal.pone.0120517 (PMC4368540; doi:10.1371/journal.pone.0120517)

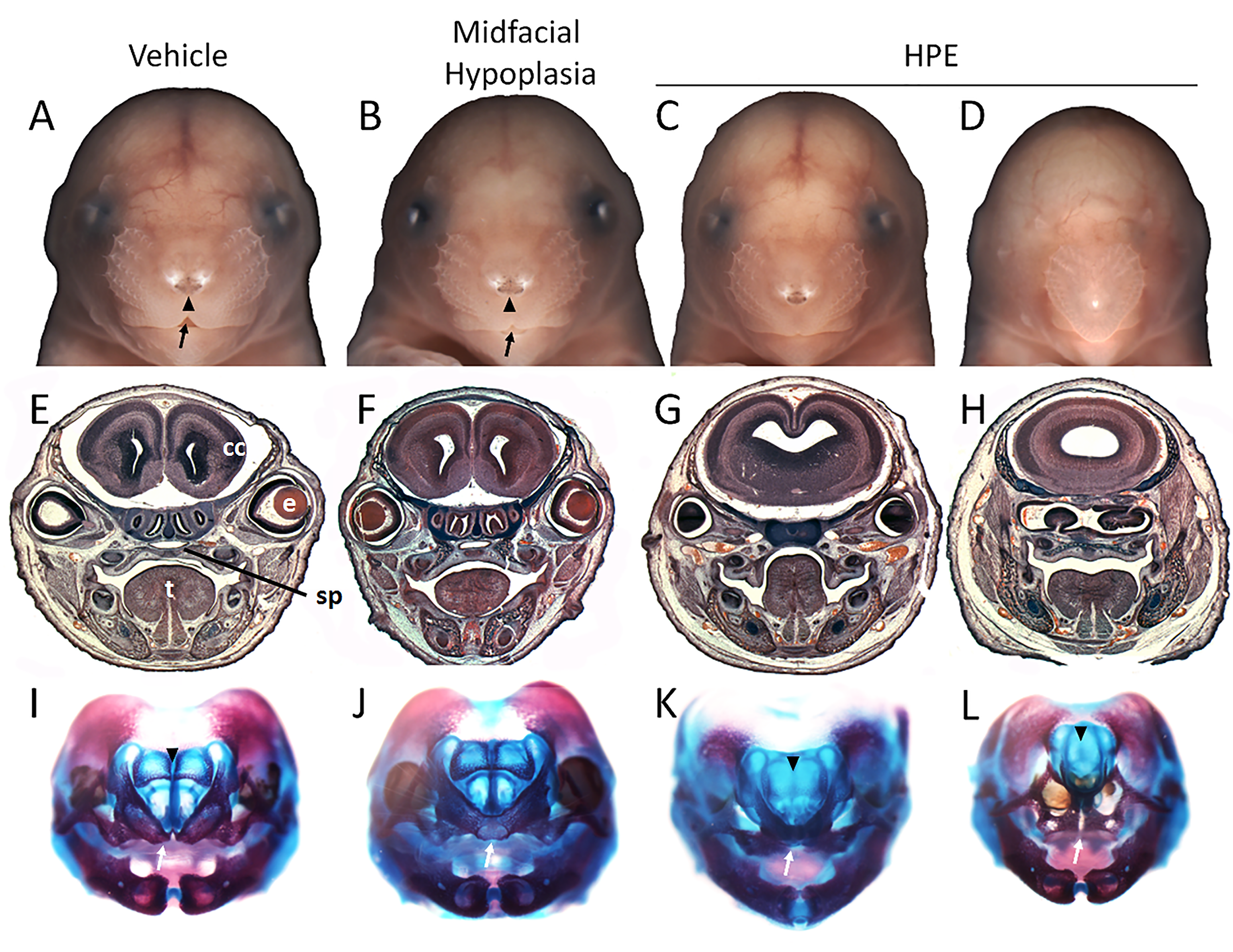

Supplement: S1 Fig — Relative to the vehicle-exposed control (A), a vismodegib-exposed fetus classified as having midfacial hypoplasia exhibits diminished area of pigmentation below the nose (arrowhead) and diminished but intact medial lip notch (B; arrow). Representative of those classified as HPE, the vismodegib-exposed fetuses shown in C and D exhibit loss of the medial lip notch with diminished or absent pigment at the tip of the nose, with the latter having a single nostril (cebocephaly). Histological sections from comparable animals show normal division of the cerebral cortices (cc) in animals classified as having midfacial hypoplasia, while those included as HPE demonstrate incomplete division of the cerebral cortices with communicating lateral ventricles. Note that the animal with HPE shown in (G) also has a secondary palate (sp) cleft. I-L show bone and cartilage staining in similarly classified animals, demonstrating a subtle midline deficiency in animals with midfacial hypoplasia, and a more severe phenotype in animals with HPE. White arrows show the premaxilla, which are fused in J, K and L. Arrowheads indicate the nasal bones, which are fused in K and L with an underlying single nasal capsule (L). (t) Tongue (e) eye. (TIF) [file pone.0120517.s001.tif]

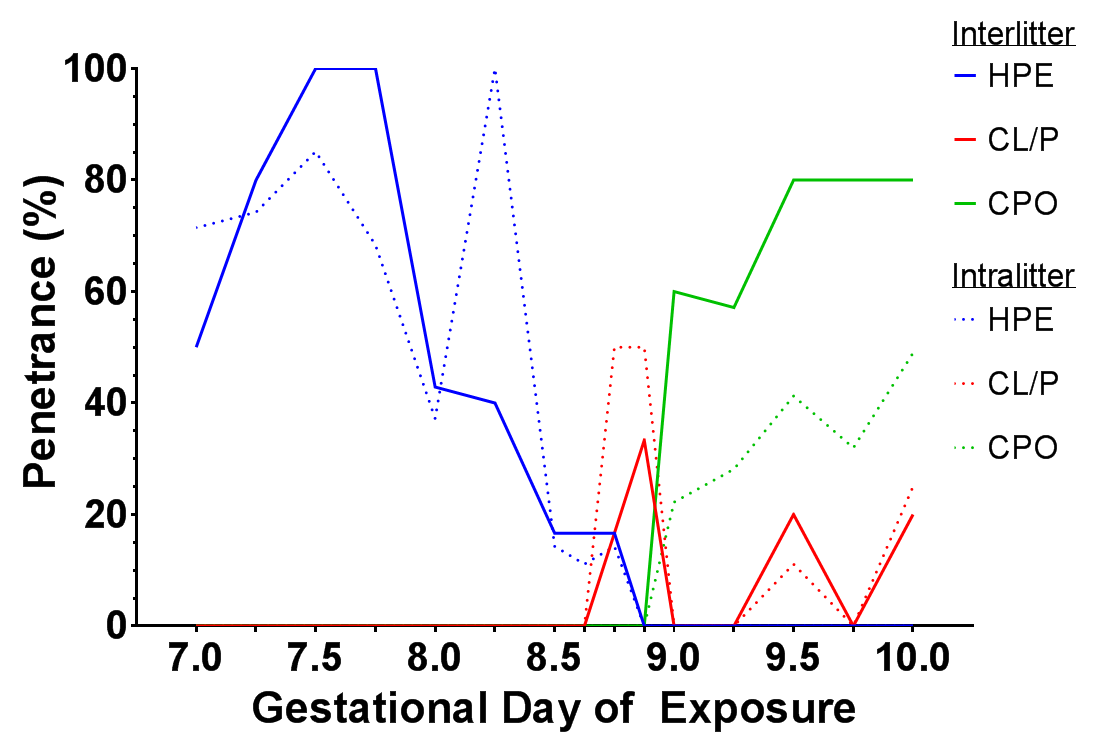

Supplement: S2 Fig — Vismodegib was administered at discrete time points indicated by tick marks on the x-axis including GD7.0, 7.25, 7.5, 7.75, 8.0, 8.25, 8.5, 8.625, 8.75, 8.875, 9.0, 9.25, 9.5, 9.75, and 10.0. Interlitter penetrance was determined by calculating the percentage of litters in which at least one affected fetus was observed. Intralitter penetrance was determined by calculating the percentage of affected fetuses within litters with at least one affected individual. (TIF) [file pone.0120517.s002.tif]

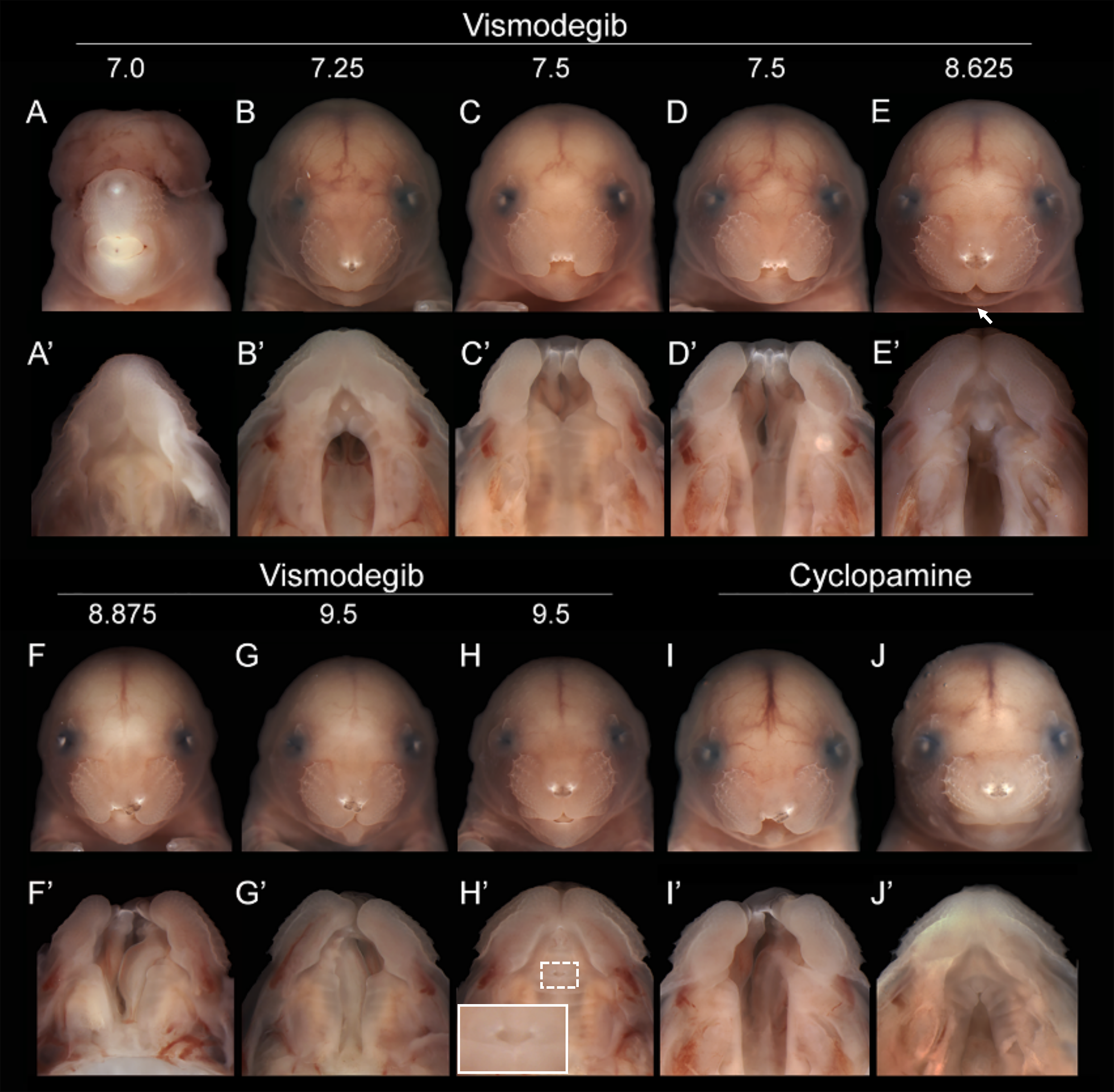

Supplement: S3 Fig — Face and palate dysmorphology infrequently co-occurring with HPE included exencephaly (A), secondary palate cleft (B, B’), median cleft lip (C, C’), or median cleft lip extending into the secondary palate (D, D’). Also infrequently, vismodegib exposure caused clefts of the secondary palate associated with severe mandibular hypoplasia (E, E’; arrow¬¬¬), cleft lip partially extending into the secondary palate, (F, F’), or incomplete cleft lip with secondary palate cleft (G, G’). Exposure during the critical period for CPO was also associated with incomplete fusion between the primary and secondary palate (H’ inset). Cyclopamine exposure infrequently caused unilateral clefts of the lip extending into the primary and secondary palate (I, I’), or midfacial hypoplasia with CPO¬ (J’). (TIF) [file pone.0120517.s003.tif]

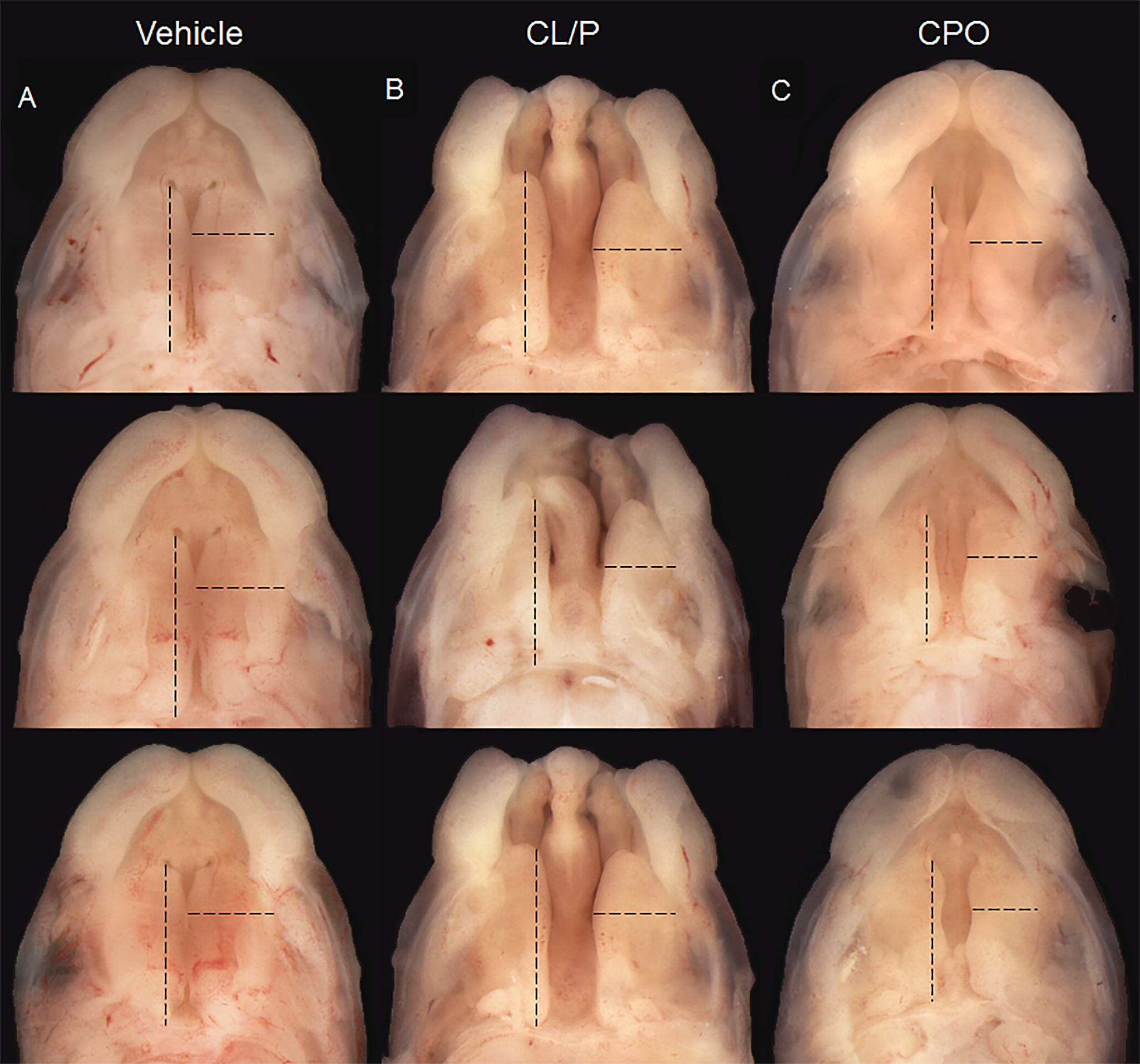

Supplement: S4 Fig — Representative GD14.5 embryos exposed to vehicle at GD9.75 (A), cyclopamine (B), or vismodegib at GD9.75 (C). Vertical and horizontal dashed lines illustrate representative linear measurements of palate shelf length and width, respectively. (TIF) [file pone.0120517.s004.tif]

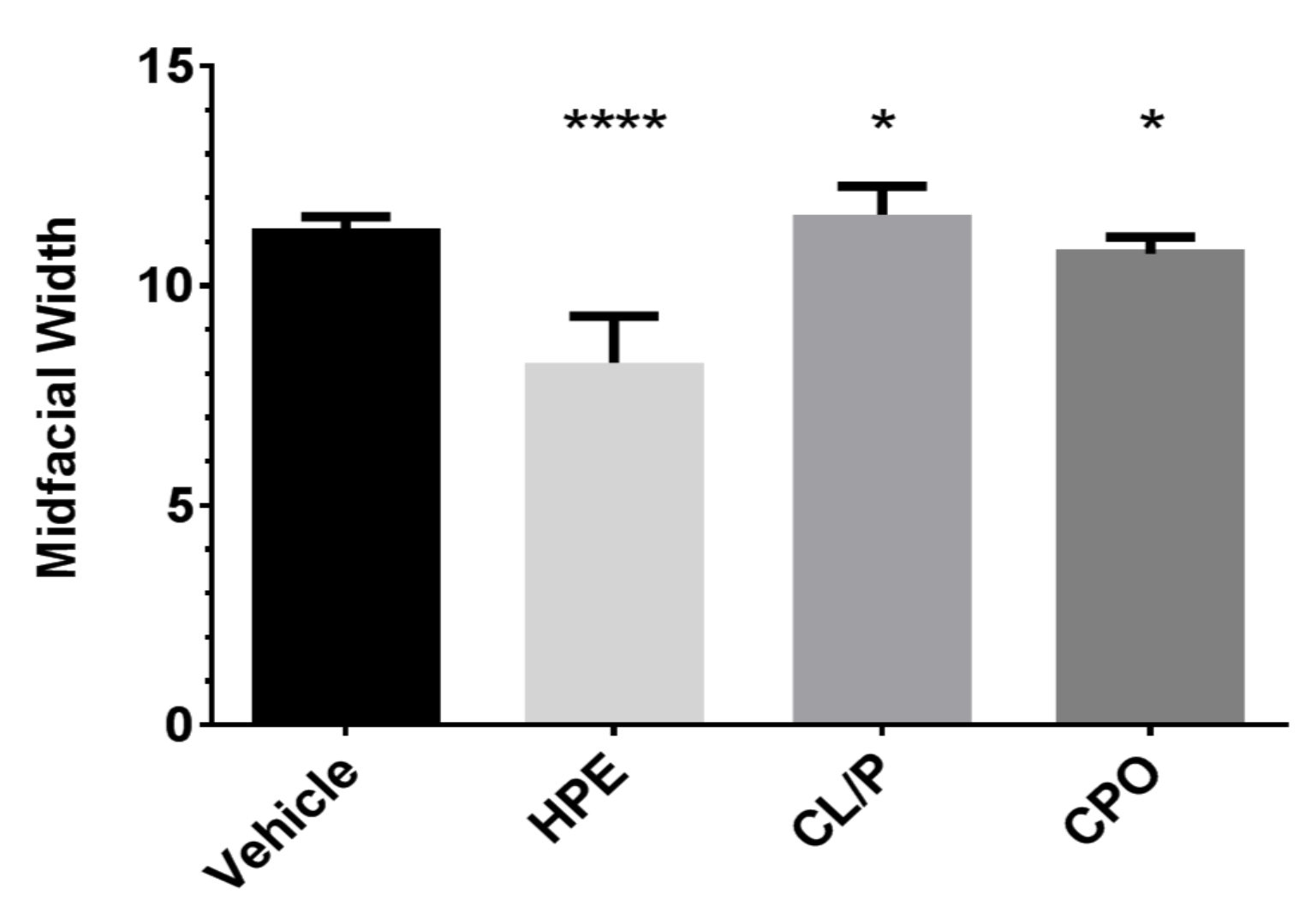

Supplement: S5 Fig — Relative to vehicle control animals, snout width is increased in animals with cyclopamine-induced CL/P, but decreased in those with vismodegib-induced HPE and CPO. Values represent the mean + S.E.M. * p<0.05, **** p<0.001, compared to vehicle-exposed control group. Units are arbitrary. (TIF) [file pone.0120517.s005.tif]

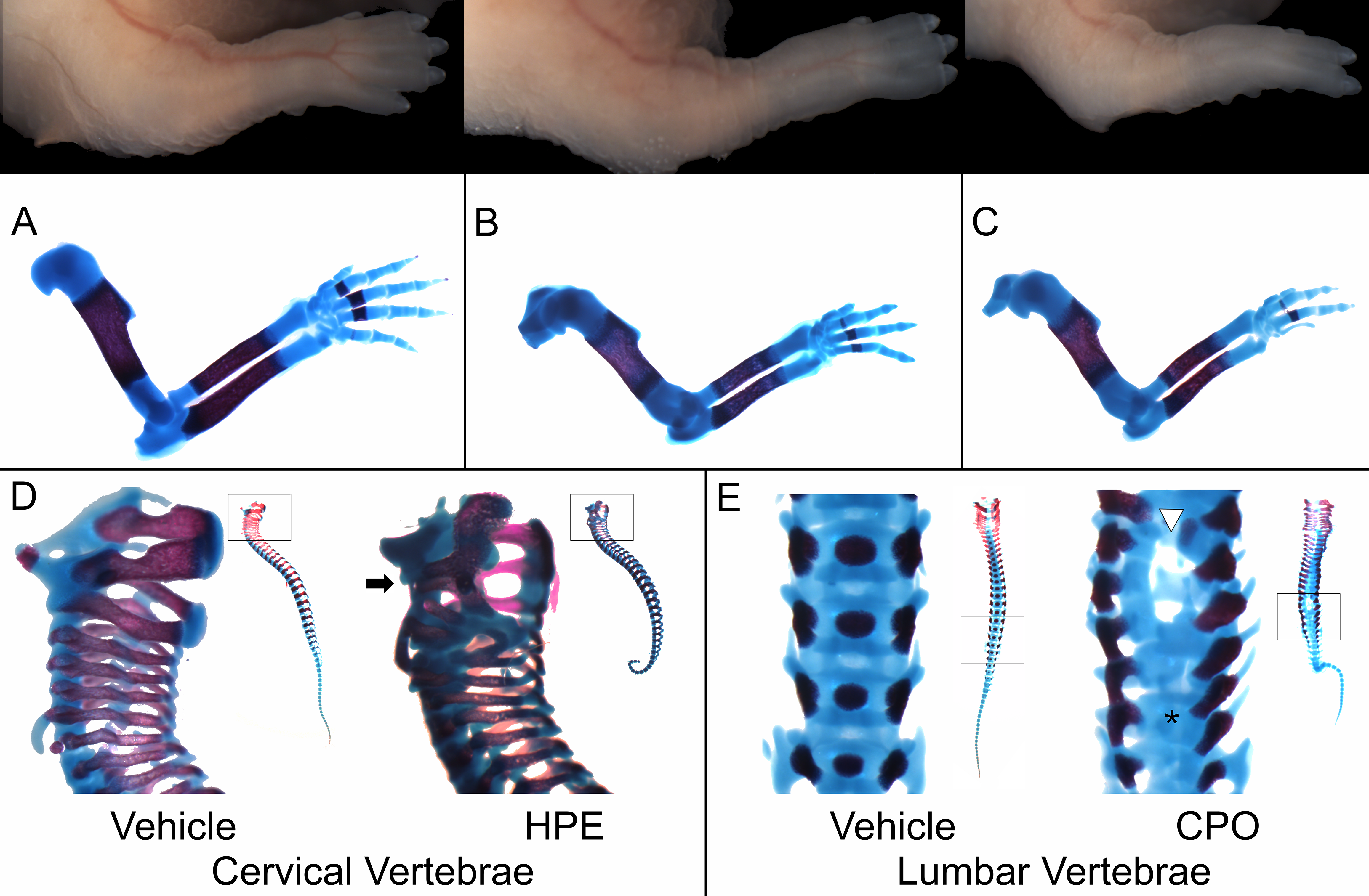

Supplement: S6 Fig — Right forelimbs of animals with vismodegib-induced forelimb abnormalities (B,C) are shown with a vehicle-exposed control (A). Varying severity of ectrodactyly, including complete absence of the fifth digit (B) and the complete absence of the fifth and partial absence of the fourth digit (C). Relatively early treatment with vismodegib resulted in HPE with accompanying cervical spine malformations including fusion of the first and second vertebrae (D; black arrow). Later treatment with vismodegib caused CPO with abnormalities in the lumbar vertebrae and cartilage (E; white arrowhead). Additionally, absent ossification in the vertebral bodies (asterisk) is apparent. Bone and cartilage are stained red and blue, respectively. (TIF) [file pone.0120517.s006.tif]

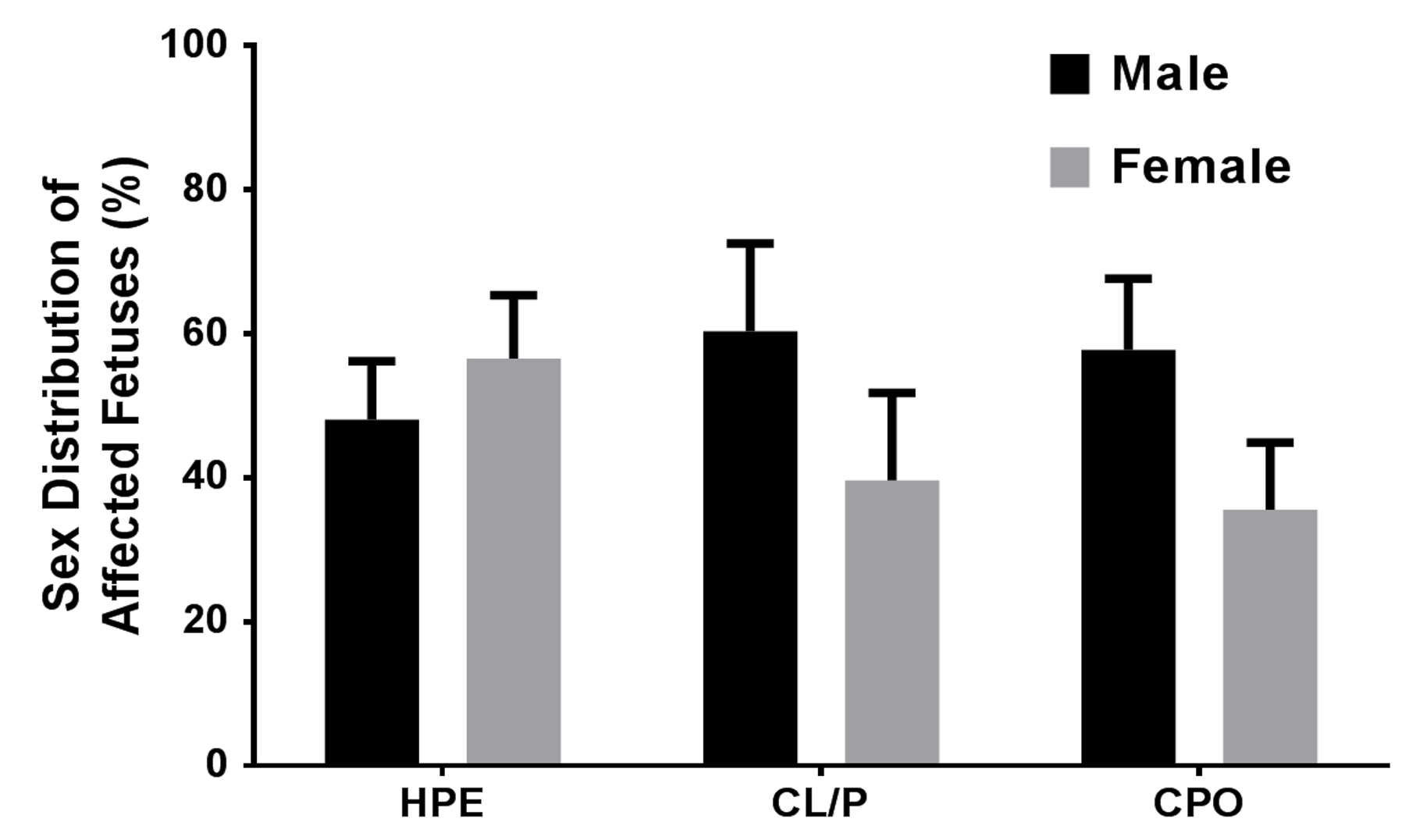

Supplement: S7 Fig — To avoid litter bias, male-female sex ratios of affected animals were determined for each litter. Values represent the mean sex distribution for HPE, CL/P or CPO + SEM. Differences were not significant by chi-square goodness of fit test (p>0.05). (TIF) [file pone.0120517.s007.tif]
